# Free-Standing Composite Film Based on Zinc Powder and Nanocellulose Achieving Dendrite-Free Anode of Aqueous Zinc–Ion Batteries

**DOI:** 10.3390/ma18122696

**Published:** 2025-06-08

**Authors:** Guanwen Wang, Minfeng Chen, Jizhang Chen

**Affiliations:** Co-Innovation Center of Efficient Processing and Utilization of Forest Resources, College of Materials Science and Engineering, Nanjing Forestry University, Nanjing 210037, China; xiangyu_163568_com@163.com (G.W.); chenmf@njfu.edu.cn (M.C.)

**Keywords:** aqueous zn–ion batteries, zinc powders, self-standing electrodes, nanocellulose utilization, dendrite suppression

## Abstract

Aqueous zinc–ion batteries (AZIBs) have garnered considerable attention owing to their inherent safety, cost-effectiveness, and promising electrochemical performance. However, challenges associated with Zn metal anodes, such as dendrite formation, corrosion, and hydrogen evolution, continue to impede their widespread adoption. To overcome these limitations, a flexible and self-standing composite film anode (denoted ZCN) is engineered from a synergistic combination of Zn powder, nanocellulose, and carbon fiber to serve as a high-performance alternative to conventional Zn foil. These three constituents play the roles of enhancing the active area, improving mechanical properties and electrolyte affinity, and establishing a conductive network, respectively. This innovative design effectively mitigates dendrite growth and suppresses parasitic side reactions, thereby significantly improving the cycling stability of ZCN. As a result, this electrode enables the Zn//Zn cell to offer an ultralong lifespan of 2000 h. And the Zn-MnO_2_ battery with ZCN anode demonstrates remarkable performance, realizing over 80% capacity retention after 1000 cycles. This study presents a straightforward, scalable, and cost-effective strategy for the development of dendrite-free metal electrodes, paving the way for durable and high-performance AZIBs.

## 1. Introduction

In recent years, escalating environmental pollution and climate change resulting from excessive dependence on fossil fuels have driven a substantial surge in the demand for clean and sustainable energy solutions. Renewable energy sources, including solar, wind, and hydropower, have emerged as promising alternatives. Nevertheless, their inherent intermittency poses significant challenges for ensuring a reliable and continuous energy supply [1,2,3,4,5]. Among diverse energy storage technologies, lithium–ion batteries have garnered considerable attention as a leading contender [6,7]. However, their limitations, such as high price, inherent flammability, and toxicity of their electrolytes, render them less suitable for large-scale grid storage applications. In this context, aqueous zinc–ion batteries (AZIBs) utilizing mildly acidic electrolytes have emerged as a safe, cost-effective, and environmentally benign alternative, capitalizing on the abundant natural reserves of zinc and the inherent advantages of aqueous electrolytes [8,9,10,11]. Nevertheless, AZIBs face challenges associated with low reversibility of zinc metal anodes [12,13,14,15]. The existing protrusions and structural defects in conventional Zn foil anodes create thermodynamically favorable sites for Zn^2+^ nucleation and deposition, easily leading to dendrite growth [16,17,18,19,20]. These dendritic structures can potentially penetrate the separator, resulting in short circuits and compromising battery safety. Moreover, dendrite formation is frequently accompanied by detrimental side reactions, such as hydrogen evolution and the generation of electrochemically inactive byproducts, which lead to fast capacity fading in AZIBs.

To address these challenges, researchers have identified three effective strategies: modifying zinc anodes, optimizing electrolytes, and establishing new separators [21,22,23,24,25]. Extensive efforts have been devoted to exploring various materials, including reduced graphene oxide, porous carbon, TiO_2_, and porous nano-CaCO_3_, as coatings on Zn foil to achieve uniform current distributions, suppress dendrite growth, and mitigate side reactions [18,26]. For instance, Zhi et al. demonstrated the effectiveness of a porous nano-CaCO_3_ coating on Zn foil, which not only prevented Zn foil corrosion but also significantly inhibited the formation of large dendrites [27]. This approach resulted in a modified Zn-MnO_2_ battery exhibiting a high capacity retention of 86% after 1000 cycles. In this study, a free-standing anode, designated as ZCN, is developed through a straightforward vacuum filtration method. This anode comprises a homogeneous integration of Zn powders (ZPs), carbon fibers (CFs), and nanofibrillated cellulose (NFC), forming a structurally unified composite film. Notably, the ZCN anode eliminates the requirement for supplementary conductive frameworks during fabrication and operation. This design synergistically leverages a three-dimensional (3D) interconnected CF network, the large surface of ZP, and the good mechanical properties and hydrophilicity of NFC, therefore enhancing ZCN’s resilience against dendrite formation during prolonged plating/stripping cycles. This endows ZCN with exceptional electrochemical stability in AZIB applications. The Zn//Zn cell with ZCN electrodes exhibits ultralong cycling durability, maintaining stable voltage profiles for 2000 h. When paired with a MnO_2_ cathode, the ZCN-based battery achieves 139.1 mAh g^−1^ after 1000 cycles at 1 A g^−1^. This study provides a scalable paradigm for next-generation metal anode engineering in aqueous batteries.

## 2. Materials and Methods

### 2.1. Preparation of NFC

Soybean straws were cleaned and then cut into small fragments, which were immersed in a mixed aqueous solution of 2.5 M NaOH and 0.4 M Na_2_SO_3_ for 5 h, and then maintained at 130 °C for 10 h. The product was washed three times with deionized (DI) water and then immersed in 2.5 M H_2_O_2_ aqueous solution, which was then heated in an oil bath at 110 °C for 4 h. After that, the product was washed three times with DI water and then freeze-dried to obtain cellulose. Approximately 0.2 g cellulose was dispersed into 400 mL aqueous solution containing 0.033 g 2,2,6,6-tetramethylpiperidine-1-oxyl radical (TEMPO) and 0.33 g of NaBr, followed by the addition of 21.3 g NaClO solution (10%). The pH of the reaction system was monitored and maintained at approximately 10 by adding 0.5 M NaOH. After 6 h, 1 mL ethanol was added to terminate the reaction, followed by five rounds of washing with DI water. Then, the washed product was subjected to ultrasonic treatment using a cell disruptor (300 W) at 0 °C for 30 min, resulting in the formation of NFC.

### 2.2. Fabrication of ZCN Films

To prepare ZCN721, 350 mg ZP (<10 μm, ≥98%, Aldrich, St. Louis, MO, USA), 100 mg CF (~100 nm in diameter, 20–200 μm in length, Aldrich), and 50 mg NFC were dispersed into 60 mL DI water using ultrasonication and stirring. The resulting dispersion was vacuum filtered through a filter membrane with a pore size of 0.22 μm. The film was then washed once with 5% HCl solution (to remove the surface passivation layer) and three times with DI water. After that, the film was peeled off from the filter membrane, freeze-dried, and pressed at 10 MPa. With a mass ratio of 7:2:1 for ZP:CF:NFC, the resulting film is named ZCN721. Accordingly, ZCN811 and ZCN631 films were prepared by a same process except for the difference in the mass ratio of ZP:CF:NFC, i.e., 8:1:1 and 6:3:1 for these two films, respectively.

### 2.3. Characterization and Electrochemical Measurements

The samples were characterized by the Rigaku Ultima IV X-ray diffractometer (XRD), Rigaku, Tokyo, Japan and JEOL JSM-7600F field emission Scanning Electron Microscope (FE-SEM), JEOL Ltd., Tokyo, Japan. A carbon nanotube (CNT)/MnO_2_ cathode material was synthesized according to a strategy proposed by Zhi et al. [27]. For the preparation of cathodes, a mixture of CNT/MnO_2_, carbon black, and polyvinylidene fluoride with a mass ratio of 7:2:1 was dispersed and thoroughly mixed in N-methylpyrrolidone, before being pasted onto a Ti foil. After drying at 80 °C for 6 h, the obtained Ti foil was pressed at 10 MPa and then cut into round pieces as the cathodes. For electrochemical measurements, CR2016-type coin cells were assembled using ZCN anode, CNT/MnO_2_ cathode, glass fiber separator, and a mixed aqueous solution containing 2 M ZnSO_4_ and 0.2 M MnSO_4_ as the electrolyte. Approximately 250 μL electrolyte was added to each coin cell. In this configuration, the mass loadings of ZP and CNT/MnO_2_ are approximately 27.9 mg cm^−2^ and 1.2 mg cm^−2^ in the anode and cathode, respectively, correpsonding to an areal capacity of 0.31 mAh cm^−2^ for the cathode and a negative/positive capacity ratio of 73.8. Galvanostatic charge/discharge (GCD) tests were carried out on CT2001A battery testing system (LANHE, Wuhan, China). Cyclic voltammetry (CV) and electrochemical impedance spectroscopy (EIS) measurements were performed on a VSP-300 electrochemical workstation (Bio-Logic, Grenoble, France).

## 3. Results

**Figure 1:**
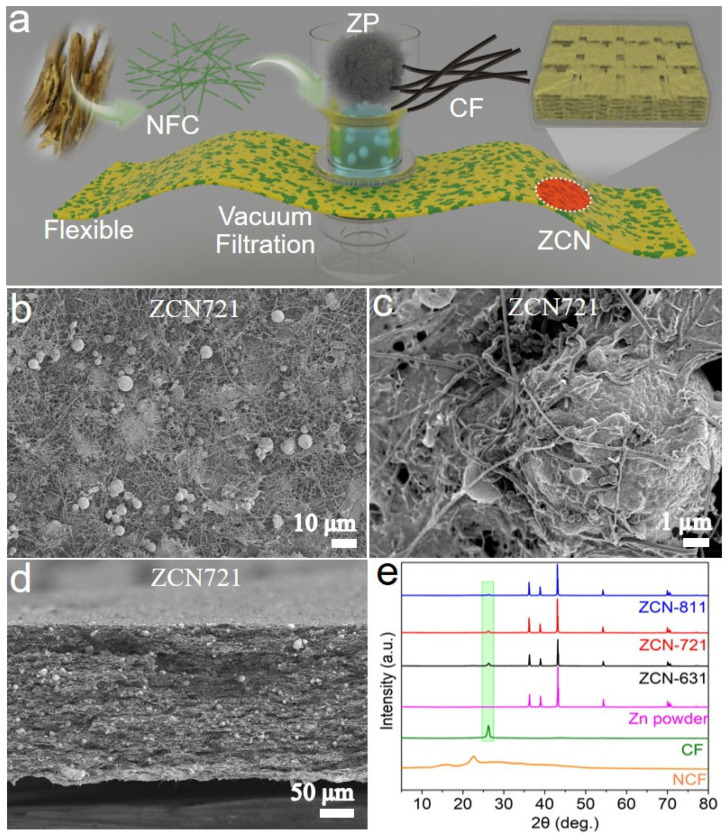
(**a**) Schematic illustration of the fabrication process of ZCN film. (**b**,**c**) Top-view and (**d**) cross-sectional SEM images of ZCN721 film. (**e**) XRD patterns of NFC, CF, ZP, and different ZCN films.

Figure 1a schematically illustrates the fabrication process of ZCN film. The process begins with soybean stalks as the raw material, from which cellulose was extracted and subsequently converted into NFC through a TEMPO-mediated oxidation strategy [28]. The obtained NFC was mixed with ZP and CF in specific ratios, followed by ultrasonication and stirring to achieve a homogeneous dispersion. The resulting mixture underwent vacuum filtration to form ZCN films, ensuring uniform distribution of these components and the creation of a flexible, self-supporting structure. After filtration, the films were washed with acidic solution and DI water, freeze-dried, and compressed at 10 MPa to enhance their structural integrity. Within ZCN films, NFC, ZP, and CF function as the mechanical scaffold, active material, and conductive agent, respectively. Owing to the merits of excellent hydrophilicity and water absorbing capability, NFC can also serve as an electrolyte reservoir and promote the transportation of Zn^2+^ ions. The morphology of the ZP is presented in Figure S1, revealing spherical shapes with diameters ranging from 2 to 10 μm. Additionally, the SEM images of CF are displayed in Figure S2, demonstrating a fiber shape with low diameter. Such morphology enables CF to greatly enhance the electrical conductivity of ZCN films. It is anticipated that the optimal ZCN film can address the challenges associated with traditional Zn foil anodes in AZIBs by leveraging the synergistic effects of its components.

As depicted in the top-view and cross-sectional SEM images of ZCN721 film (Figure 1b–d), ZP, CF, and NFC are uniformly distributed, forming a dense and compact film. Compared to conventional Zn foils, ZPs can provide a significantly larger electrochemical active area, hence promoting homogeneous zinc plating/stripping. Notably, the ZPs are tightly wrapped by CFs (Figure 1c), a unique architectural design that further enhances the stability of ZCN721 film. Additionally, the voids among these components provide additional space to accommodate the deposited Zn, enhancing the film’s capacity and structural stability during electrochemical cycling. As shown in Figure 1d, the thickness of ZCN721 film is approximately 200 μm. The XRD patterns of ZCN films and their constituents are presented in Figure 1e. Due to the low crystallinity of NFC, its presence in ZCN films cannot be detected by XRD. However, the XRD patterns of ZCN films reveal a small hump arising from CF and a series of intense peaks attributed to ZP (i.e., metallic Zn). As the amount of CF added decreases sequentially from ZCN631 to ZCN721 and then to ZCN811, the XRD peaks from CF exhibit a gradual attenuation in intensity. These results underscore the successful integration of three components and the structural integrity of ZCN films.

**Figure 2:**
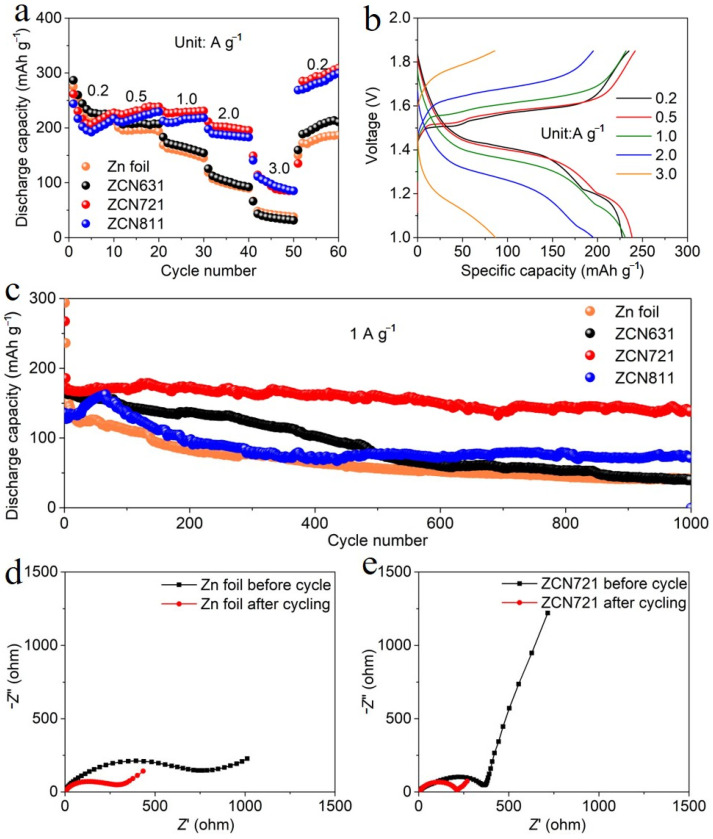
(**a**) Rate performances of Zn-MnO_2_ batteries with different anodes. (**b**) GCD curves of the Zn-MnO_2_ battery with ZCN721 anode at different current densities. (**c**) Cycling performances of Zn-MnO_2_ batteries with different anodes at 1 A g^−1^ (pre-cycled twice at 0.2 A g^−1^). Nyquist plots of the Zn-MnO_2_ batteries before and after cycling: (**d**) with Zn foil anode and (**e**) with ZCN721 anode.

Given the exceptional combination of high operating potential and large specific capacity offered by MnO_2_ cathode materials [29,30], we employed a CNT/MnO_2_ nanocomposite as the cathode material in this study. As illustrated in Figure S3, except for the peak at around 26° ascribed to CNT, all the XRD peaks of the CNT/MnO_2_ nanocomposite can be indexed to α-MnO_2_ (JCPDS: 44-0141). Coin-cell-type AZIBs were assembled using CNT/MnO_2_ cathode and different anodes, and their performances were evaluated at current densities from 0.2 to 3 A g^−1^ (Figure 2a). Among different ZCN films, ZCN721-based battery demonstrates the best rate capability. Specifically, the Zn-MnO_2_ battery with ZCN721 anode delivers a discharge capacity of 227.4 mAh g^−1^ at 0.2 A g^−1^ (based on the mass of CNT/MnO_2_ and measured at the last cycle of each current density). This capacity gradually decreases to 238.5, 230.8, 195.4, and 85.1 mAh g^−1^ as the current density increases to 0.5, 1, 2, and 3 A g^−1^, respectively. Notably, when the current density was restored to 0.2 A g^−1^, a capacity of 308.8 mAh g^−1^ can be realized at the 60th cycle. Furthermore, the capacities achieved with ZCN721 anode surpass those obtained with Zn foil anode, particularly under high-rate conditions and during prolonged cycling.

The superior rate of performance of the Zn-MnO_2_ battery with ZCN721 anode can be attributed to the following aspects. First, the 3D conductive network established by CFs ensures efficient and uninterrupted electronic transport across the electrode with reduced local current density [31,32]. Second, the uniform distribution of ZPs embedded within the film provides a high density of accessible active sites for Zn^2+^ stripping/plating, promoting rapid ion diffusion and minimizing kinetic limitations [18]. Third, the NFC serves a dual role: it not only acts as a robust binder to maintain mechanical integrity but also contributes to the formation of a well-defined porous architecture, enhancing electrolyte infiltration and optimizing ionic transport pathways [33,34,35]. These synergistic features collectively reduce polarization and improve charge transfer kinetics, particularly at elevated current densities. In contrast, Zn foil anode is prone to uneven Zn deposition and dendrite formation, which increase internal resistance and accelerate capacity degradation under high-rate operation. The GCD profiles of the Zn-MnO_2_ battery with ZCN721 anode are depicted in Figure 2b. At a current density of 0.2 A g^−1^, two distinct discharge plateaus are observed, with a turning point at approximately 1.3 V. These two plateaus correspond to proton intercalation (higher potential) and Zn^2+^ ion intercalation (lower potential), respectively [36]. Even at 1 A g^−1^, the electrochemical polarization remains at a rather low level, highlighting efficient electrochemical behavior of ZCN721 film.

The cycling performances of Zn-MnO_2_ batteries with different anodes were evaluated at 1 A g^−1^, as shown in Figure 2c. The battery with Zn foil anode exhibits the worst cyclability, primarily due to dendrite formation and parasitic reactions. Among all the ZCN anodes, the employment of ZCN721 demonstrates the best cycling stability. Specifically, the battery with ZCN721 anode retains a capacity of 139.1 mAh g^−1^ at the 1000th cycle, corresponding to 81.4% of its capacity at the 3rd cycle of 1 A g^−1^. And the GCD curves at different cycles exhibit good overlap in the shape (Figure S4), further confirming good electrochemical reversibility. In stark contrast, the capacity retention of the battery with Zn foil anode is only 23.6% after 1000 cycles under the same conditions. Given that the utilization of ZCN721 film contributes to the best rate capability and cycling performance, subsequent discussions will focus exclusively on this electrode. To further investigate the difference in electrochemical kinetics and interfacial stability when using ZCN721 and Zn foil, EIS measurements were carried out before and after long-term cycling, and the obtained Nyquist plots are presented in Figure 2d and Figure 2e, respectively, with the equivalent circuit presented in Figure S5. Before cycling, the Zn foil electrode exhibits a relatively large semicircle in the high-frequency region, corresponding to a high charge transfer impedance (*R*_ct_) of 739.9 Ω. After 1000 cycles, the *R*_ct_ decreases to 287.8 Ω, which can be attributed to the increased surface roughness and expanded surface area caused by uncontrolled dendrite growth and side reactions. However, despite the reduced *R*_ct_, the performance of the Zn-MnO_2_ battery with Zn foil anode deteriorates dramatically, as previously shown in Figure 2c. In contrast, the use of ZCN721 electrode displays a much lower initial *R*_ct_ of 365.3 Ω, indicative of faster kinetics at the electrode/electrolyte interface. After 1000 cycles, the *R*_ct_ further decreases to 215.7 Ω, reflecting the formation of a stable and conductive interface during cycling. This reduction in impedance, combined with the excellent capacity retention, confirms that ZCN721 anode facilitates uniform Zn deposition and suppresses the accumulation of insulating byproducts.

**Figure 3:**
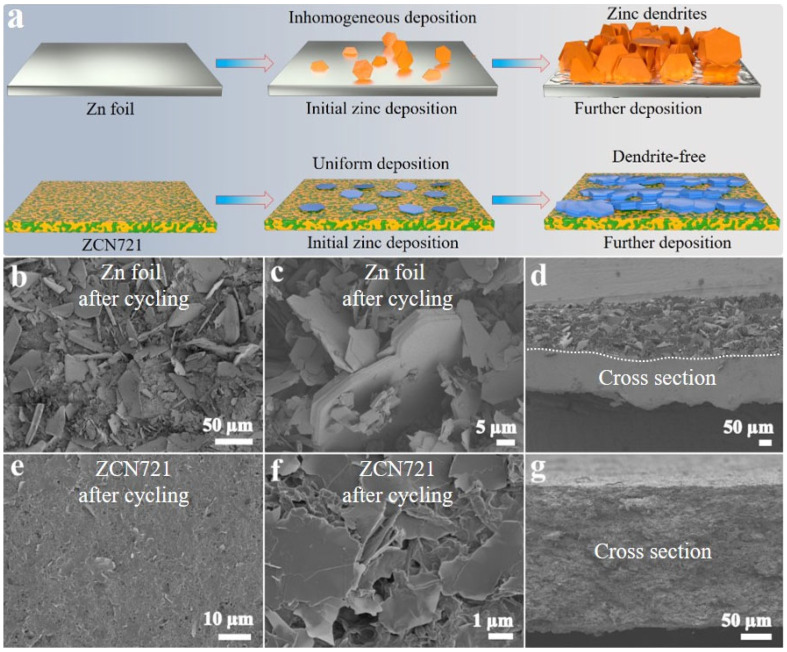
(**a**) Illustration of the morphological evolution of Zn foil and ZCN during the zinc deposition process. (**b**,**c**,**e**,**f**) Top-view and (**d**,**g**) cross-sectional SEM images of (**b**–**d**) Zn foil and (**e**–**g**) ZCN721 electrode after 1000 cycles.

Figure 3 illustrates morphological evolution of the zinc surface after cycling, comparing Zn foil with ZCN721 electrode. As depicted in Figure 3a, the Zn foil exhibits inhomogeneous zinc deposition at the initial stage, leading to uncontrolled dendrite growth in the later stages of zinc deposition. In stark contrast, ZCN721 enables highly uniform zinc deposition. In this configuration, the use of CF can establish a 3D interconnected network for fast electron transport and homogeneous current distribution, while NFC provides superior hydrophilicity and zincophilicity. Therefore, dendrite formation can be effectively suppressed. To gain deeper insights into the morphological changes of Zn foil and ZCN721 after long-term cycling, SEM characterization was conducted. After 1000 cycles, the Zn foil displays a rough surface adorned with large and irregular zinc protuberances (Figure 3b,c), indicative of severe dendrite growth. The cross-sectional view in Figure 3d reveals a thick and uneven deposited layer, which is related to unstable Zn plating/stripping behavior. Conversely, the ZCN721 electrode retains a significantly flatter surface after prolonged cycling (Figure 3e,f). Only minor surface roughness is observed, with no apparent dendritic structures, suggesting uniform and horizontal zinc deposition. Figure 3g illustrates a smooth cross-section of the ZCN721 electrode after long-term cycling, devoid of noticeable inhomogeneous deposition layer, further attesting to its good dendrite-suppressing efficacy.

**Figure 4:**
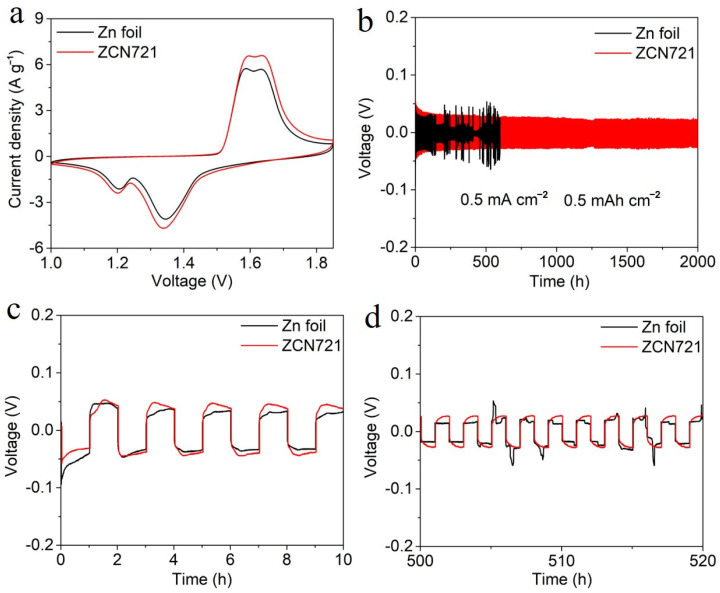
(**a**) CV curves of Zn-MnO_2_ batteries with Zn foil and ZCN721 as the anodes at 0.5 mV s^−1^. (**b**) Cycling performances of Zn//Zn cells with Zn foil electrodes or ZCN721 electrodes at 0.5 mA cm^−2^ and 0.5 mAh cm^−2^. GCD profiles of Zn//Zn cells with Zn foil electrodes or ZCN721 electrodes: (**c**) during the initial 10 h and (**d**) during 500–520 h.

Figure 4a presents CV curves of Zn-MnO_2_ batteries with ZCN721 or Zn foil anode at a scan rate of 0.5 mV s^−1^. The battery with ZCN721 anode exhibits a higher peak current compared to that with Zn foil anode, indicative of superior reaction activity of ZCN721 anode. This phenomenon aligns with the previously demonstrated high capacity when utilizing ZCN721 anode. To further validate good electrochemical reversibility of ZCN721 film, Zn//Zn symmetric cells were subjected to long-term cycling at an areal capacity of 0.5 mAh cm^−2^ and a current density of 0.5 mA cm^−2^, with the outcomes depicted in Figure 4b. The ZCN721-based cell sustains a remarkably stable and flat voltage profile for 2000 h with minimal voltage variations. Such lifespan surpasses that of Zn//Zn cells involving various modified Zn electrodes in previous reports (under the same or milder test condition), as compared in Table S1. In striking contrast, the Zn foil-based cell displays an unstable voltage profile characterized by near-zero potential difference during charge and discharge in some regions and pronounced voltage fluctuations, implying dendrite formation and side reactions that impair performance. To clearly demonstrate the voltage behavior across different cycling phases, the GCD curves of Zn//Zn cells during the initial 10 h and during 500–520 h of cycling are shown in Figure 4c and Figure 4d, respectively. The ZCN721-based cell retains much smoother and more symmetrical profiles than the Zn foil-based cell, further verifying ZCN721’s remarkable electrochemical stability [37].

## 4. Conclusions

In summary, a free-standing Zn powder–carbon fiber–nanocellulose composite film is engineered as a high-performance anode for aqueous zinc–ion batteries to address challenges of dendrite formation, corrosion, and hydrogen evolution when using conventional Zn foil anode. The optimized ZCN721 film, with its tailored component ratios, can enhance the active surface area, improve mechanical robustness and electrolyte affinity, and establish a conductive network for efficient electronic transfer by synergistically utilizing the advantages of Zn powder, nanocellulose, and carbon fiber. This innovative architecture effectively mitigates dendrite growth and suppresses parasitic reactions, significantly enhancing the cycling stability of ZCN721 electrode. This electrode can enable the Zn//Zn cell to achieve an ultralong lifespan of 2000 h, demonstrating exceptional durability. Furthermore, when paired with a MnO_2_ cathode, the ZCN721-based battery exhibits remarkable performance, retaining over 80% of its capacity at the 1000th cycle. This work not only presents a simple, scalable, and cost-effective strategy for the development of dendrite-free zinc anodes but also underscores great potential of composite film architectures in advancing next-generation batteries.

## Data Availability

The original contributions presented in this study are included in the article/Supplementary Material. Further inquiries can be directed to the corresponding author.

## Supplementary Materials

The following supporting information can be downloaded at: https://www.mdpi.com/article/10.3390/ma18122696/s1, Figure S1: SEM images of ZP with different magnifications.; Figure S2: SEM images of CF with different magnifications.; Figure S3: XRD pattern of CNT/MnO_2_ cathode material.; Figure S4: GCD profiles of the Zn-MnO_2_ battery with ZCN721 anode at the 3rd and 1000th cycles of 1 A g^−1^; Figure S5: Equivalent circuit used in this work. Table S1. Comparison of the lifespan of Zn//Zn cell with ZCN721 electrodes in this work with that of Zn//Zn cells involving various modified Zn electrodes in previous reports. References [38,39,40,41,42,43,44,45,46,47] have been cited in the Supplementary Materials.
