# Supplementary material for: Free-Standing Composite Film Based on Zinc Powder and Nanocellulose Achieving Dendrite-Free Anode of Aqueous Zinc–Ion Batteries
Source: Materials (Basel). 2025 Jun 8;18(12):2696. doi: 10.3390/ma18122696 (PMC12194234; doi:10.3390/ma18122696)
Supplement: Supplementary file 1 [file materials-18-02696-s001.zip › materials-3596146-supplementary.pdf]

Supporting Information

Free-standing composite film based on zinc powder and nanocellulose achieving dendrite-free anode of aqueous zinc-ion batteries

Guanwen Wang[a], Minfeng Chen[a], Jizhang Chen[a,*]

[a] *Co-Innovation Center of Efficient Processing and Utilization of Forest Resources, College of Materials Science and Engineering, Nanjing Forestry University, Nanjing 210037, China*

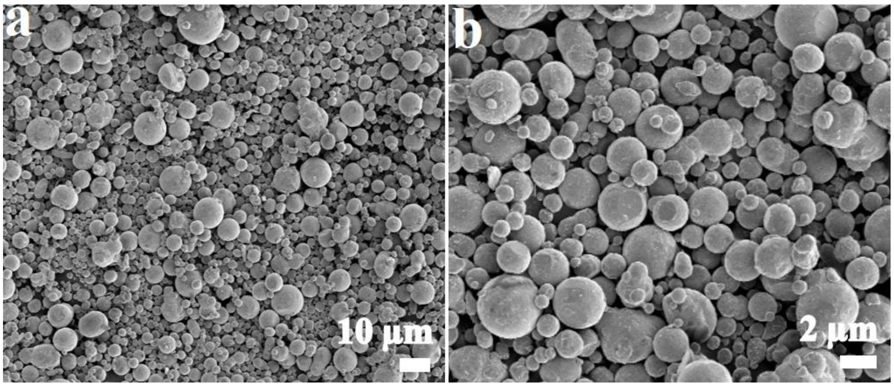

Fig. S1. (a, b) SEM images of ZP with different magnifications.

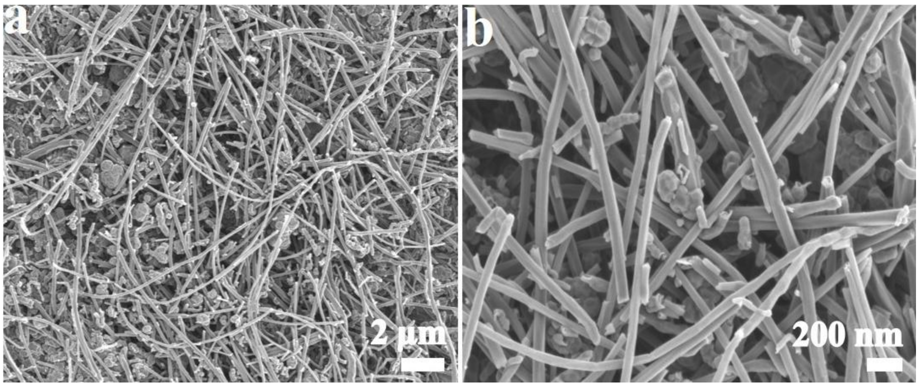

Fig. S2. (a, b) SEM images of CF with different magnifications.

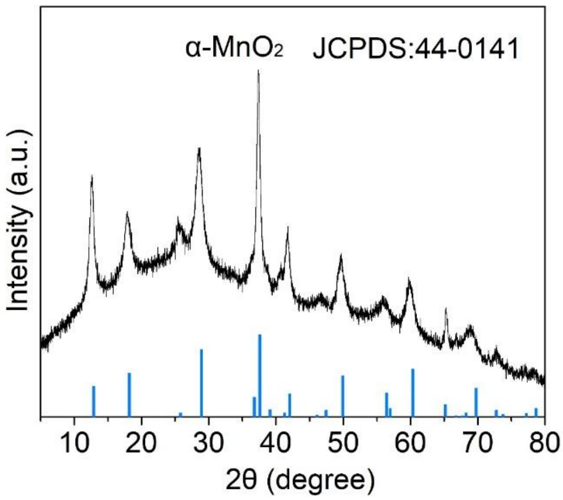

Fig. S3. XRD pattern of CNT/MnO_2 cathode material.

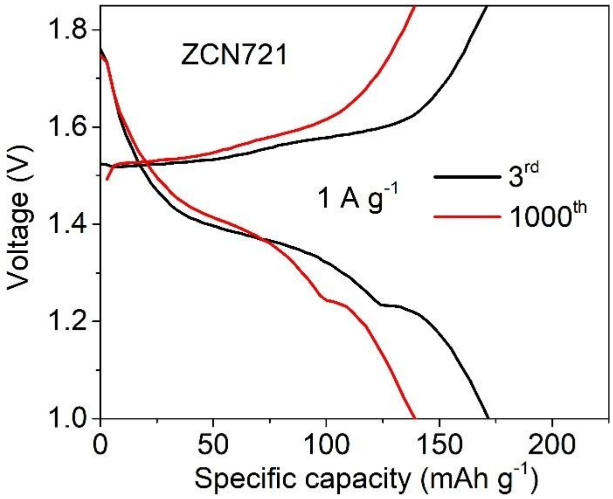

Fig. S4. GCD profiles of the Zn-MnO_2 battery with ZCN721 anode at the 3rd and 1000th cycles of 1 A g^{-1}.

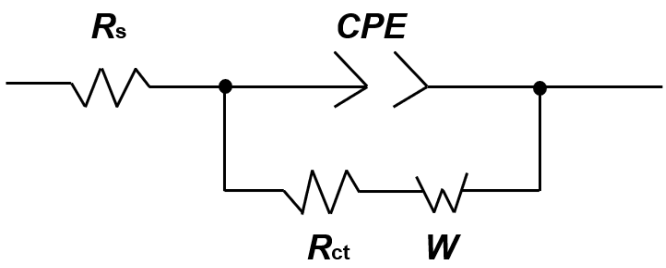

Fig. S5. Equivalent circuit used in this work.

Table S1. Comparison of the lifespan of Zn//Zn cell with ZCN721 electrodes in this work with that of Zn//Zn cells involving various modified Zn electrodes in previous reports.

Zn electrode	Current density	Areal capacity	Lifespan	Ref.
ZCN721	0.5 mA cm^{-2}	0.5 mAh cm^{-2}	2000 h	This work
Zn@P-CN	0.5 mA cm^{-2}	0.5 mAh cm^{-2}	900 h	[1]
ZnTAPP-TFTA@Zn	0.5 mA cm^{-2}	0.5 mAh cm^{-2}	1100 h	[2]
ISNF@CNTs@Zn	0.5 mA cm^{-2}	0.5 mAh cm^{-2}	1500 h	[3]
3D MXene array@Zn	0.5 mA cm^{-2}	0.5 mAh cm^{-2}	1350 h	[4]
ZnOHF@Zn	0.2 mA cm^{-2}	0.2 mAh cm^{-2}	1000 h	[5]
Zn|LM	0.5 mA cm^{-2}	0.5 mAh cm^{-2}	800 h	[6]
Zn-InF_3	0.5 mA cm^{-2}	0.5 mAh cm^{-2}	1400 h	[7]
NVP-Ga(0.5)@Zn	0.5 mA cm^{-2}	0.5 mAh cm^{-2}	1300 h	[8]
Zn*x*Cu*y*/Zn	0.5 mA cm^{-2}	0.5 mAh cm^{-2}	<2000 h	[9]
Zn-1D-1 min	0.5 mA cm^{-2}	0.5 mAh cm^{-2}	1100 h	[10]

References

1. Xiao, Y.; Su, T.; Wang, T.; Xiang, W.; Tang, S.; Yu, J. S., Interfacial micro-electric field induced by phosphorus-doped g-C3N4 for highly reversible dendrite-free zinc metal anode. *Chemical Engineering Journal* **2025**, 512, 162391.
2. Shen, P.; Pu, X.; Zhang, X.; Liu, Y.; Han, D.; Sun, X.; Wang, H.-g., A porphyrin-functionalized conjugated microporous polymer coating for stable dendritic-free aqueous zinc ion batteries. *Chemical Engineering Journal* **2024**, 493, 152440.
3. Dong, J.; Duan, J.; Cao, R.; Zhang, W.; Fang, K.; Yang, H.; Liu, Y.; Shen, Z.; Li, F.; Liu, R.; Jin, M.; Lei, L.; Li, H.; Chen, C., Dendrite-free Zn deposition initiated by nanoscale inorganic–organic coating-modified 3D host for stable Zn-ion battery. *SusMat* **2024**, 4, e189
4. Ruan, J.; Ma, D.; Ouyang, K.; Shen, S.; Yang, M.; Wang, Y.; Zhao, J.; Mi, H.; Zhang, P., 3D artificial array interface engineering enabling dendrite-free stable Zn metal anode. *Nano-Micro Letter* **2023**, 15, (1), 37.
5. Liang, Y.; Kou, Y.; Hao, Q.; Chen, F.; Chen, X.; Li, N., Improving Zn ion transport behavior and uniform deposition using artificial ZnOHF coated film for deeply rechargeable Zn metal anodes. *Electrochimica Acta* **2023**, 443, 141928.
6. Liu, C.; Li, Z.; Zhang, X.; Xu, W.; Chen, W.; Zhao, K.; Wang, Y.; Hong, S.; Wu, Q.; Li, M. C.; Mei, C., Synergic effect of dendrite-free and zinc gating in lignin-containing cellulose nanofibers-mXene layer enabling long-cycle-life zinc metal batteries. *Advanced Science* **2022**, 9, (25), e2202380.
7. Zhou, Y.; Tong, H.; Wu, Y.; Chen, X.; Wu, C.; Xu, Z.; Shen, L.; Zhang, X., A dendrite-free Zn anode Co-modified with In and ZnF_2 for long-life Zn-ion capacitors. *ACS Applied Materials & Interfaces* **2022**, 14, (41), 46665–46672.
8. Huang, X.; Cao, H.; Liu, Y.; Hu, Q.; Zheng, Q.; Zhao, J.; Lin, D.; Xu, B., Na superionic conductor-type compounds as protective layers for dendrites-free aqueous Zn-ion batteries. *Journal of Colloid and Interface Science* **2023**, 629, 3–11.
9. Meng, H.; Ran, Q.; Dai, T. Y.; Shi, H.; Zeng, S. P.; Zhu, Y. F.; Wen, Z.; Zhang, W.; Lang, X. Y.; Zheng, W. T.; Jiang, Q., Surface-alloyed nanoporous zinc as reversible and stable anodes for high-performance aqueous zinc-ion battery. *Nano-Micro Letter* **2022**, 14, (1), 128.
10. Wu, Z.; Zou, J.; Li, Y.; Hansen, E. J.; Sun, D.; Wang, H.; Wang, L.; Liu, J., Regulating zinc nucleation sites and electric field distribution to achieve high-performance zinc metal anode *via* surface texturing. *Small* **2023**, 19, (5), e2206634.
